# Insights from molecular dynamics simulation of human ceruloplasmin (ferroxidase enzyme) binding with biogenic monoamines

**DOI:** 10.6026/97320630015750

**Published:** 2019-10-31

**Authors:** Bishnu Prasad Mukhopadhyay

**Affiliations:** 1 Department of Chemistry National Institute of Technology-Durgapur, West Bengal, Durgapur-713209, India

**Keywords:** Ceruloplasmin, biogenic monoamines, conserved water molecules

## Abstract

Human ceruloplasmin (hCP) is a multi-copper oxidase with ferroxidase and amine oxidase activities. Molecular dynamics simulation (MDS) and docking analysis of biogenic monoamines with ceruloplasmin explain the role of Asp1025, Glu935, Glu272, Glu232 and Glu230 together with the binding site water molecules (referred as conserved water molecules) in the stabilization of neurotransmitter (Serotonin, Norepinephrine and Epinephrine) molecules within the binding cavity of hCP. Conserved water molecules are found at specific positions interacting with the protein structures that have sequence similarity. The ethylamine side chain nitrogen atom (N1) of neurotransmitter molecules interacts with water molecules in the binding cavity formed by Asp1025, Glu935 and Glu232 residues. These residues form an acidic triad mimicking a substrate binding cavity. The hydroxyl groups attached to the catechol ring of epinephrine and norepinephrine have been stabilized by Asp230 and Asp232 residues. Data suggests that the recognition of biogenic amines mediates through the N+(amine) ...Asp1025-His1026-Cu_Cis-His_ path. The potential recognition path of biogenic monoamines to trinuclear copper cluster supported by active site water molecules (referred as conserved water molecules) is described in this report.

## Background

Ceruloplasmin (hCP) is one of the most complex blue multi copper oxidases which is most popular and least understood metallo enzymes [1]. Human CP is a monomer having 1046 residues with a carbohydrate content 7-8% and few crystallographic structures are available in Protein Data Bank at different resolution from 2.6 to 3.1 Å [2-4]. In contrast to three domain structure of ascorbate oxidase, bilirubin oxidase and laccase containing one type1 copper ion and a trinuclear copper cluster, CP has six copper ions and is comprised of six compact beta barrel domains with large loop insertions. The enzyme contains three mononuclear T1- copper centers (Cu_PR_, Cu_RS_ and Cu_Cys-His_) in domains 2, 4, and 6, with the separation of ∼18 Å, whereas the other three copper (T2/T3) centers form a trinuclear cluster which is situated at the interface between domains 1 and 6 [3]. Again interaction of conserved water molecules with the trinuclear copper(T2/T3) cluster and role of water molecules in the recognition of three T1 mononuclear copper centers have also been indicated in the MD-simulation studies of hCP [5,6]. The enzyme is involved with Wilson [7] and Menke's diseases, and associated to acerulo plasmenia [8]. It shows multifunctional activities in the physiological system e.g., ferroxidase activity [9], amine oxidase activity, antioxidant activity, inhibition of myelo peroxidase activity etc. and also involved in the copper ion transportation in plasma [10], however detail role of the metallo enzyme is still unknown. There are seven categories of inhibitors of CP: inorganic anions, chelating agents, carboxylate anions, thiol compounds, hydrazines, 5-hydroxy indoles, divalent and trivalent metal cations etc. [11]. The enzyme can also catalyze or oxidize the different substrates like biogenic monoamines, aromatic diamine and (+) lysergic acid diethylamide (LSD) and they have distinct different binding sites (at the different domains). Consequently the association of hCP in the oxidation of biogenic monoamines, like epinephrine (adrenalin), norepinephrine (noradrenalin) and serotonin (5-hydroxy tryptamine) is thought to have some importance concerning to regulation of the level of those neurotransmitters in bloodstream which could be important for brain-functions [12].The enzyme oxidizes the norepinephrine and epinephrine to adreno chrome and serotonin to 5-hydroxy indole-3-acetic acid [11]. Earlier studies on the effect of drugs used in the treatment of mental illness, e.g. tranquilizers and anti-depressants have also indicated the importance of biogenic monoamines interaction with hCP. Enhanced oxidation of dopamine by hCP has also been found in Parkinson's disease [13, 14].

Conserved water molecules are thought to be an integral part of protein and they involve in the structure-function-activity [15-18], metal to metal [19, 20] or metal to substrate/ligand interaction in metallo enzymes. They also play a vital role in the stabilization and activation of a variety of integral membrane proteins [21]. Previous crystallographic studies have shown the tentative location of biogenic monoamines (binding site) near to Fe (II) binding center in domain 6 of that enzyme [22]. But until now the detail and exact oxidation mechanism of biogenic monoamines is still unknown and it requires more theoretical and experimental investigation. The present MD-simulation studies on the biogenic monoamines-ceruloplasmin complexes have provided the detail recognition mechanism of neurotransmitter to this metallo enzyme. Plausible role of acidic triad and few conserved water molecules in the binding of neurotransmitters to CP as well as the recognition of biogenic monoamines to copper center and copper cluster may also be enlightened from the investigation which might have some importance to the biology of this metallo enzyme.

## Methodology

The PDB structure 2J5W [3] was used for MD- simulation studies. Monomeric unit of CP was present in the asymmetric unit along with few ions, small organic ligands and 341 number of water molecules. In entire manuscript, the numbering scheme for six integral copper ions, amino acid residues, and water molecules kept same as were given in the crystal structure.

## Structure preparation:

The two N-acetyl-D-glucosamine (NAG) groups, oxygen atom near Cu3049, two glycerol (GOL) molecules, and an extra labile Cu2+ ion were removed from the PDB structure. Then missing residues at the different sequences 476-482 (Tyr-Asn-Pro-Gln-Ser-Arg-Ser), 885-889 (Tyr-Leu-Lys-Val-Phe) and 1042-1046 (Asp-Thr-Lys-Ser-Gly) were added in the protein structure. Then successive energy minimization of all residues was followed stepwise by steepest descent (1000 steps) and conjugate gradient (2000 steps) methods using SwissPdb viewer program. The final hCP structure was checked by superimposing it on the 2J5W PDB structure using UCSF Chimera program. The stereo chemical arrangements of the added and other residues of the different domains were also verified by Ramachandran plot. Total ∼98% of the residues of protein were within the favoured and allowed region of Ramachandran plot and no residues were observed at outliers. Few close contacts between the non-hydrogen atoms were edited properly. In the final structure there were no chirality outliers, torsion outliers and ring outliers. The bond lengths and bond angles were verified properly. In the final model (hCP) structure, the oxygen (O_2_) molecule was placed and fixed within the trinuclear copper cluster in such a way so that the two oxygen atoms could form bonds with the metal centers as was found in the X-ray structure.

## Protein–ligand docking:

Previous crystallographic studies have indicated the tentative location of biogenic monoamines (near to domain 6) in CP (PDB-structure 1KCW having resolution 3.1Å), where the resolution of epinephrine, serotonin and norepinephrine complexes were 3.2, 3.1 and 3.3Å [11].However, the resolution and electron density map did not provide the detailed and exact information on the binding sites of those neurotransmitters, and even no attempts were made to refine the binding sites due to limitations in the resolution of diffraction data sets[11]. Moreover beside the presence of six (6) integral copper ions in the enzyme, that crystal contained an extra labile Cu^+2^-ion (Cu1054) at ∼9Å away from the nearby integral T-1 copper center (Cu1053).In 2J5W PDB structure an extra labile Cu^+2^-ion (Cu3053) was also observed to occupy at the same crystallographic position of 1KCW structure [3, 4]. So for investigating the interaction of biogenic monoamines with native CP, the Cu3053 atom was removed from the 2J5W structure before the docking of neurotransmitter.

Ligand-receptor docking was separately performed using AUTODOCK VINA v.1.1.1 [23]. The 2J5W structure (excluding the water, extra labile copper ions and other ligand molecules) was considered as receptor. The PDBQT file of the receptor protein was generated using AutoDock Tools v.1.5.4 [24] by assigning Kollman united atom charges. The structures for epinephrine, norepinephrine and serotonin (protonated amine form) were converted into PDBQT file after including their partial atomic charges using Gasteiger method [25]. Grid point spacing was set at 1 Å and 20 grid points were taken in each direction. The grid box was centered at the putative location site near to domain 6. VINA automatically calculated the grid map for searching. All other docking parameters were assigned to their default values. For each ligand, the five best results of docked complexes were selected serially according to their binding affinity and the first one was chosen for further work. The docking energies for serotonin, norepinephrine and epinephrine with CP were -4.9, -4.5 and -4.6 kcal/mol. Each ceruloplasmin-neurotransmitter docking positions were also validated by SwissDock server [26].

## Identification of conserved water molecules:

The 3DSS server [27] and Swiss PDB viewer program were used to find out the conserved water molecules in the MD simulated structures. The 2J5W PDB-structure [3] was taken as reference and the other MD-simulated structures were successively superimposed on it. The cut-off distance between the pairs of superposed water molecules was taken to be 1.8 Å.

## Molecular dynamics (MD) simulation:

**Figure 1:**
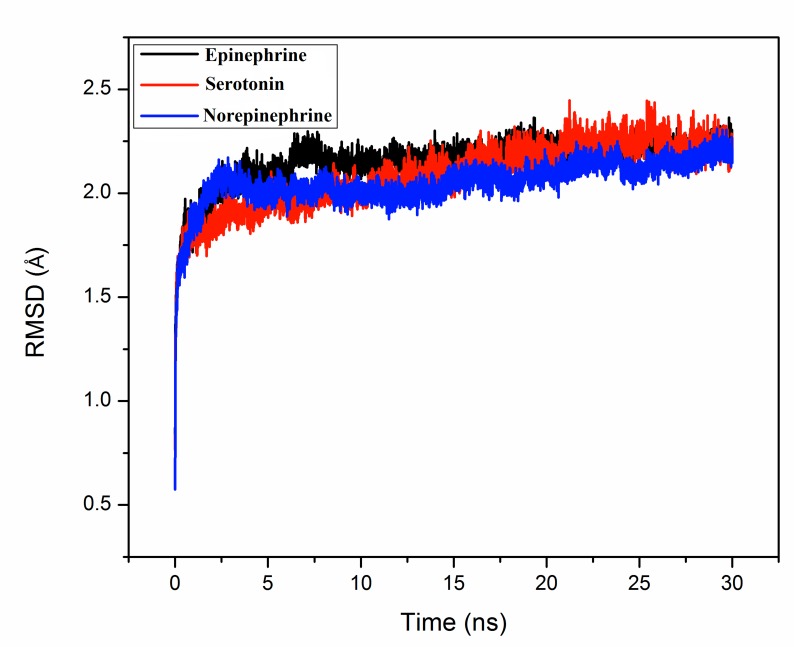
The RMSD (root mean square deviation) curve for serotonin (red), norepinephrine (blue) and epinephrine (black) during MD-simulation of their complexes with ceruloplasmin

MD-simulation of all the structures were performed using NAMD v.2.6 [28] with CHARMM36 force field [29]. The charges for the six integral copper atoms and oxygen molecule (Cu3046:0.7932, Cu3051: 0.7101, Cu3052: 1.0356, Cu3047: 1.4302, Cu3048: 1.4861, Cu3049: 1.4154, O1: - 0.5068 and O2: - 0.5301) of the enzyme and neurotransmitter (serotonin, epinephrine and norepinephrine) molecules were obtained from quantum chemical and NBO calculations performed on the truncated optimized structures of hCP following the previous protocols [5], and the charges were included on the respective atoms and molecules before the simulation of neurotransmitter-CP complexes. All copper atoms of the trinuclear cluster (Cu3047, Cu3048, and Cu3049) and T1 mononuclear centers (Cu3046, Cu3051, Cu3052) were kept fixed. Before running the simulation constrains was applied on the copper coordinated atoms of His, Cys and Met residues. Then each structure was converted to Protein Structure File (PSF) by Automatic PSF Generation Plug-in within VMD program v. 1.9.3 [30]. The crystal water molecules were retained and converted to TIP3P water model. All the Na^+^ and Ca^+2^ ions present in the crystal-structure were also retained and included in simulation. Energy minimization of each docked complex was performed in two successive stages; initial energy minimization was performed for 1000 steps by fixing the backbone atoms, followed by a final minimization for 2000 steps (conjugate gradient) considering all the atoms of system to remove residual steric clashes. The energy-minimized structures were then simulated at temperature (310 K) and pressure (1 atm) by Langevin dynamics using periodic boundary condition. The Particle Mesh Ewald method was applied for full-electrostatics and Nose-Hoover Langevin piston method used to control the pressure and dynamical properties of the barostat. At the initial stage of simulation, water dynamics was performed for 2 ns by fixing the protein residues and allowing the water molecules to move freely within the ligand-docked structure. Finally, all-atom molecular dynamics simulation was performed for total 90ns, in which 30 ns for each biogenic monoamine bound ceruloplasmin structure. All the docked-structures were equilibrated within ∼5ns. During simulation the atomic coordinates were recorded at every 2 ps interval for analysis. The root mean square deviation (RMSD) of the three simulated complex structures were calculated and shown in Figure 1. The simulation trajectory was analysed from 1 to 30ns to investigate the interaction of neurotransmitters with hCP.

## Results and Discussion:

MD-simulation studies of biogenic monoamines-ceruloplasmin complexes have provided some new insights on the interaction of those ligands which were not explored in the crystallographic investigations. During simulation all the three neurotransmitter molecules (serotonin, norepinephrine and epinephrine) have occupied in the cavity (proturburence) present on the upper surface of hCP near to T1 copper center (Cu3052) of domain 6 and stabilized through hydrogen bond network of several acidic residues and few conserved/ semi conserved water molecules. Hydrogen bonding interaction and distances of the potential sites of three biogenic monoamines (SER (serotonin),NOR (norepinephrine) and EPI (epinephrine)) from the residues and water molecules at different time are given in Table 1.

## Stabilization of neurotransmitters in the binding cavity of CP:Serotonin

During simulation, serotonin molecule adapts trans-conformation, the torsion angles χ1 (C12-C4-C3-C2) and χ2 (C4-C3-C2-N1) are varied from 70 to 113 ° and -135 to -166 °. At different time the protonated amino nitrogen (N1+) atom of serotonin is observed to interact with the residues Asp1025, Glu935 and Glu272of an acidic triad present in the neurotransmitter binding cavity of CP and also interacts with a conserved water (W_S_1) center with occupation frequency (O.F.)∼ 100% (Table 1).

**Figure 2:**
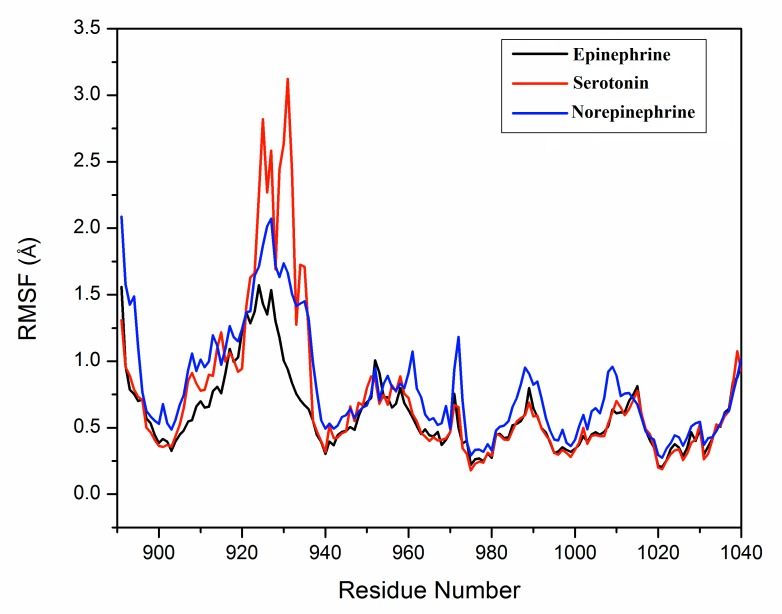
RMSF (root mean square fluctuation) curve of the residues (sequence 900-1040) of ceruloplasmin during MD-simulation of enzyme-biogenic monoamine complexes.

During the entire period , the helix containing the residue Asp1025 is observed to interact with side chainN1-atom of serotonin and the Asp1025(OD2)...N1 distance is ranging from 2.56 to 2.68 Å, and Glu272 of a loop interacts with that nitrogen center upto 18.04ns, where the distance is varied from 2.50 to 2.71Å, however after that period, that glutamic acid residue is stabilized by His1026 (Nb) atom through H-bond ,upto ∼ 18ns Glu935(OE1) is stabilized by salt bridge interaction with Lys938(NZ) where the distance varied from 2.56 to 2.78 Å, and the distance of this acidic residues to side chain N1 atom of serotonin was found to be ∼7 Å. But after18.04ns, the helix (consisting of seven residues from Glu931 to Asn937) is moved away from that position due to disruption of salt-bridge, consequently Glu272...N1 interaction decreases and then Glu935 (of that helix) come close to the side chain N1-atom of serotonin, where the distance varied from 2.54 to 2.87 Å.During simulation the high RMSF values of that helix (residues from 931 to 937) have also indicated the high fluctuating tendency of Glu935 residue which was shown in Figure 2.

**Figure 3:**
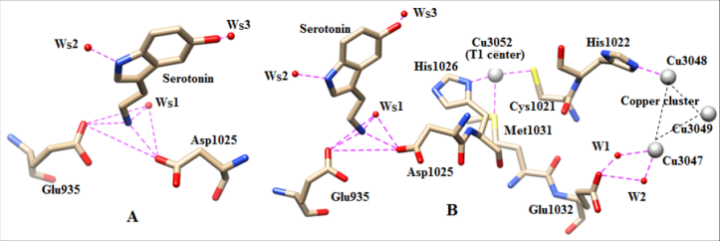
(A).The H-bonding interaction of serotonin with the three conserved water molecules (W_S_1, W_S2_ and W_S_3), Asp1025 and Glu935 is shown. (B) The recognition of serotonin to Cu (T1) center. Direct and water mediated recognition path of serotonin to trinuclear copper center via Cu (T1) center. The nitrogen and oxygen centers have indicated by blue and red colours. The water molecules have shown by red balls

After ∼5ns, the N6-nitrogen atom of serotonin is stabilized by Glu232(OE1) bound semi conserved water molecule WS2(O.F. ∼85%). The N6...WS2 and WS2...Glu232(OE2) distances are ranging from 2.60 to 2.91 and 2.78 to 3.09 Å.The hydroxyl group (O10) of heterocyclic ring interacts withAsn271(ND2) andforms H-bond with another conserved water molecule (WS3) having ∼ 100% residential frequency. The WS3...O10, Asn271 (ND2)...O10 and Asn271(ND2)...WS3 distances are ranging from 2.47 to 2.77, 3.10 to 3.50 and3.0 to 3.25 Å (Table 1). The WS3 water center also forms H-bond with another acidic residue Asp206(OD2) with O.F. ∼90% and the distance ranges from 2.50 to 3.03 Å. It is interesting that Glu232 has been stabilized by salt-bridge with Arg239 through H-bonds.

upto ∼15.5ns, where the OE1...NH1 and OE2...NH2 distances were ranging from 2.6 to 3.2 and 2.5 to 3.1 Å. However, after ∼ 17ns,the salt-bridge pattern has been changed, where the residue Asp206 was observed to participate in the interaction with Arg239(NH2) through Glu232(OE1)...(NH1)Arg239(NH2) ...Asp206 (OD2) hydrogen bonds, where the Glu232 (OE1)... Arg239(NH1) and Asp206(OD2) ...Arg239(NH2) distances were 2.56 to 2.76 and 2.68 to 3.11 Å respectively. It is interesting to note that the heterocyclic indole ring is stabilized by a salt-bridge mediated H-bond chain through conserved water molecules: N6(serotonin)... W_S_2...Glu232 ...Arg239 ... Asp206 ... W_S_3...O10. In the serotonin complexed MD structure, the ethylamine side chain N+-bound WS1 site is observed to present near the position of labile Cu2+-ion (Cu3053) present in the 2J5W crystal structure. The stabilization of serotonin by network of H-bonds with the residues and water molecules is shown in Figure 3A.

## Norepinephrine:

**Figure 4:**
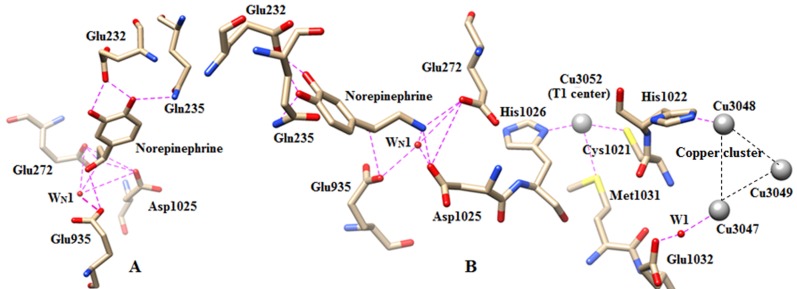
(A).The H-bonding interaction of norepinephrine with the conserved water molecule, Asp1025 and Glu935. (B) The recognition of norepinephrine to Cu (T1) center. Direct and water mediated recognition path of serotonin to trinuclear copper center via Cu (T1) center. The nitrogen and oxygen centers have indicated by blue and red colours. The water molecules have shown by red balls.

During simulation of ceruloplasmin-norepinephrine complex, the torsion angles χ1(C5-C4-C3-C2) and χ2(C4-C3-C2-N1) of norepinephrine (NOR) vary from ∼78.33 to ∼ -164.94° and ∼149.32 to ∼163.45° and it adapts trans conformation. The side chainethylamine N1-nitrogen atom of norepinephrine interacts with the Asp206, Asp1025 and a conserved water molecule (WN1). However, after ∼7ns onward, Asp206(OD2) forms salt bridge with Arg239(NH2) where the distance is ranging from 2.67 to 3.28 Å ,then the nitrogen atom has been stabilized by Asp1025(OD1), Glu272(OE2) and WN1 through H-bonds. The ethylamine side chain N+-bound WN1 hydrophilic site is found near to labile Cu2+ (Cu3053) bound water center W2327 (of 2J5W crystal structure). The benzylic hydroxyl group (O3 atom) of norepinephrine interacts with the different acidic residues of the binding cavity e.g., Asp1025(OD2) or Glu272(OE2) or Glu935(OE2) at different time through H-bonds with different residential frequencies (Table 1). During simulation, initially the OD2 atom of Asp1025 interacts to hydroxyl O3 atom, but after some time (∼6ns) Glu272 interacts with that hydroxyl oxygen, however sometimes one water molecule (W2326) was also found to stabilize that hydroxyl group through H-bond. Again the Glu935 forms salt-bridge with Lys938 upto∼15ns, but after ∼20ns that acidic residue forms hydrogen bond with O3-atom of norepinephrine. The O3...Asp1025, O3...Glu272(OE2) and O3...Glu935(OE2) distances are ranging from 2.82 to 2.88, 2.45 to 2.62 and 2.51 to 2.94 Å. The catechol ring attached hydroxyl groups (O7 and O8 atoms) have been stabilized by carboxyl oxygen atoms of Glu232 through H-bonds which were given in Table 1. The stabilization of norepinephrine by different residues and water molecules is shown in Figure 4A. Similar type of interaction between the beta hydroxyl group with water molecule has also been seen in the crystal structure of norepinephrine-Phenyl ethanolamine N-methyl tranferase (PNMT) complex (PDB Id. 3HCD) and further QM/MM studies were also indicated the water mediated interaction of N+-site to acidic residues of protein. Recent MD-simulation and DFT studies have also indicated the role of conserved water molecules on the binding of some neurotransmitter molecules like dopamine and phenyl ethylamine at the active site of human Monoamine oxidase B [31, 32] where these water molecules stabilized the amino terminal and catechol hydroxyl groups by hydrogen bond interaction.

## Epinephrine:

**Figure 5:**
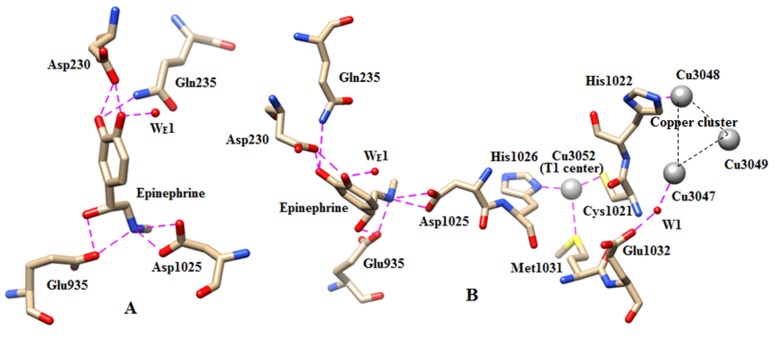
(A) The H-bonding interaction of epinephrine with the conserved water molecule, Asp1025 and Glu935. (B) The recognition of epinephrine to Cu (T1) center. Direct and water mediated recognition path of serotonin to trinuclear copper center via Cu (T1) center have shown in the right side of the figure. The nitrogen and oxygen centers have indicated by blue and red colours. The water molecules have shown by red balls.

During simulation of the complex, epinephrine adapts trans- conformation and the torsion angles χ1 (C5-C4-C3-C2) and χ2 (C4-C3-C2-N1) of neurotransmitter vary from 54.56 to 77.27 and 165.44 to 174.72 °. At different time of simulation, the side chain amino N1- atom of epinephrine interacts with the Asp1025(OD1/OD2) and Glu935(OE1/OE2) residues through H- bonds with ∼100% and ∼84% O.F. and the distances are ranging from ∼2.56 to 3.41 and ∼2.55 to 3.23 Å. After ∼6.6ns, Glu935 of a helix (residue no. 931-937) interacts with both the amino-nitrogen and β-hydroxyl group of epinephrine through H-bonds. The benzylic hydroxyl group (O3 atom) forms H- bonds with Asp1025 and a water molecule (W2051) upto∼6ns where the O3...Asp1025 (OD2) and O3...W distances are varied from 2.60 to 2.82 and 2.73 to 2.94 Å. But after that period (∼7ns) Glu935(OE1/OE2) has stabilized the O3-atom through H- bond (O.F.∼80%)where the distances were varied from ∼2.64 to 2.76 Å. The para-hydroxyl O7- atom of catechol ring is stabilized by Asp230(OD1) and a conserved water (WE1) center (∼100% O.F.) , where O7...Asp230 and O7...WE1 distances are ranging from ∼2.50 to 2.76 and ∼2.74 to 3.16 Å. The meta-OH group (O8 atom) of catechol ringforms H-bond with Asp230(OD1/OD2) with ∼100% O.F., however sometime Gln235(NE2) was also observed to interact with that oxygen center of epinephrine. The O8...Asp230(OD1/OD2) and O8...Gln235(NE2) distances are ranging from ∼2.57 to 2.70 and ∼3.20 to 3.45 Å respectively (Table 1). Throughout the simulation, the aromatic phenyl group of epinephrine has observed to stabilize by T-shaped π...π interaction with Phe209 through it was not been observed in norepinephrine-CP complex. Stabilization of epinephrine by the acidic residues and water molecules has been shown in Figure 5A.

## Acidic triad in the neurotransmitter binding cavity:

In CP, the biogenic monoamine binding cavity is lined by several (∼6-7) acidic residues which are playing crucial role in the binding of neurotransmitter. In the biogenic monoamine- ceruloplasmin complexes the functional groups of neurotransmitters are mostly stabilized by acidic residues and few conserved/ semi conserved water molecules through H-bonding interaction. The presence of conserved water molecules have also been observed in the active site of different proteases [33], kinases [34], transferases [17] and other enzymes [35] which are thought to be important for their catalytic functions and to bind the ligands and substrates molecules to the proteins. Generally, in CP the side chain N1-amino group of biogenic monoamines are interacting with the three acidic residues Asp1025, Glu935 and Glu272 with different O.F. ranges from ∼50 to 100%. It is interesting to note that these three acidic residues are spatially oriented within the neurotransmitter binding cavity in such a way so that they form a triad like geometry, where the average distances ofAsp1025(OD1)...Glu935(OE1), Asp1025(OD1)...Glu272(OE1) and Glu935(OE1)...Glu272(OE1) are ∼ 5.1, 4.7 and 6.6 Å respectively. However, in the crystal structure of uncomplexed ceruloplasmin (PDB Id: 4ENZ) the corresponding distances are 6.08, 4.05 and 7.11 Å. Thus, within the neurotransmitter binding cavity of enzyme, stereo chemical arrangement of the three acidic groups is such that they could create an environment within which the basic amino N1-nitrogen atom of monoamines been cleaved and stabilized by any two or three residues of that acidic triad.

During entire simulation period of the neurotransmitter-ceruloplasmin complexes, generally the side chain carboxyl group of Asp1025 and Glu935 are interacting with 2-3 water molecules (on an average) , however Glu272 shows lower hydration susceptibility compare to them. Moreover, Asp230 and Glu232 residues of the second domain are also oriented within the neurotransmitter binding cavity in such a way that they could allow them to make H-bond with the para or meta hydroxyl groups of catechol ring of norepinephrine and epinephrine. Similarly conserved water molecules have also been playing structural and functional role in the catalytic triad of serine [36] and cysteine proteases [37] which are composed of Ser, His, Asp and Cys, His, Asn residues respectively but in CP the triad is mainly build by three acidic residues which seem to be unique in character. So, possibly it may be presumed that the conserved water molecules and acidic triad of CP may directly or indirectly be involved in the catalysis or oxidation of neurotransmitters though the mechanism is still unknown.

## Recognition of neurotransmitter to metal centers:

Recognition between the metal centers and metal to ligand/substrate molecule is thought to be an important aspect for metallo enzyme function and activities. The degradation or oxidation of substrate (neurotransmitter) by ceruloplasmin is thought to mediate through electron transfer from a mononuclear T1 copper center (CuCys-His) to trinuclear copper cluster via the covalent-linked Cu(T1)-Cys1021-His1022-Cu (copper cluster) path followed by the reduction of O2-molecule [38]. Simulation studies of the complexes can also provide some light on the probable or possible recognition path of biogenic monoamines to copper cluster via the mononuclear copper center (Cu_Cys-His_). In all the three substrate bound enzyme complexes, the ethylamine N1-amino site of substrate recognizes the trinuclear copper cluster via a mononuclear copper center through: N+(monoamine)...Asp1025-His1026-Cu3052(T1)-Cys1021-His1022-Cu3048 (copper cluster) interaction. During simulation, the distances of N+ atom of serotonin, norepinephrine and epinephrine from the type1 Cu3052 center are ∼10.75,10.50 and 12Å respectively. Moreover, conserved water mediated recognition between the mononuclear copper bound substrate molecule to copper cluster via the acidic Glu1032 residue has also been observed in all these complexes: N+(monoamines)...Asp1025-His1026-Cu3052(T1)-Met1031-Glu 1032 ...W1/W2...Cu3047 (copper cluster), where the W1 and W2 water centers are occupied by different water molecules having high residential frequencies. In serotonin, the Met1031 bound acidic residue Glu1032 has connected the Cu3047 (of trinuclear copper cluster) through Glu1032(OE2)...W1...Cu3047 and Glu1032(OE1)...W2...Cu3047 interaction where the two hydrophilic positions have occupied by W2090 and W2338 water molecules with ∼100% O.F. However, in norepinephrine and epinephrine the interaction of Glu1032 to copper cluster have been mediated via a single water site W1 which was occupied by a water molecule W2090 with ∼100% O.F. The interaction of type1 copper center to trinuclear copper center has also been noted in the 2J5W crystal structure [3], where three water molecules were observed to connect the Glu1032 with Cu3047 of copper cluster through linear array of H-bonds (Glu1032(OE1)...W2331...W2090... W2338...Cu3047). Such interaction of Glu1032 and its communication to trinuclear copper cluster through conserved water centers have also been observed in the simulated structure of unliganded ceruloplasmin [5].Possibly the water molecules (W1/W2) play some role in the communication between Glu1032 and copper cluster and it may be important for maintaining the function of that glutamic acid residue.In fact, several studies have suggested the possible role of Glutamic acid located near to trinuclear copper cluster of multi copper oxidases (which is Glu1032 in human ceruloplasmin) to act as proton donor to the copper cluster which could be used for the reduction of O2 molecule [39]. All the results have indicated some plausible rational on the structural and functional role of some acidic residues and conserved water molecules in the interaction of biogenic monoamines to enzyme which could shade some new light on the chemistry of ceruloplasmin.

## Conclusion

The detailed recognition mechanism of neurotransmitters with the enzyme by several acidic residues and conserved water molecules is illustrated using molecular dynamics simulation and docking analysis of biogenic monoamine with the human ceruloplasmin. Data shows that Asp1025, Glu935 and Glu272 acidic residues of a triad (present within the monoamine binding cavity) have stabilized the protonated ethylamine side chain nitrogen atom (N1^+^) of serotonin, norepinephrine and epinephrine through H-bonding interaction with variable residential frequencies with a unique type of Asp1025_OE1/OE2_...N1 interaction in all the cases. The recognition of biogenic monoamines (to T1 copper center of ceruloplasmin) mediates through Asp1025 residue of domain 6: N^+^(amine)...Asp1025-His1026-Cu_Cis-His_(T1). The benzylic hydroxyl group (O3-atom) of norepinephrine and epinephrine is stabilized by Glu935 through H-bonds with high frequency of occurrence. However, Asp1025 and Glu272 also interact in few occasions. The two hydroxyl groups of catechol ring are stabilized by Glu232 (O.F. almost 100%) in norepinephrine, whereas they are stabilized by Asp230 and a conserve water molecule W_E_1 through H-bonds with almost 100% O.F (occupation frequency) in epinephrine. The potential recognition path of biogenic monoamines to trinuclear copper cluster via T1-copper centre has been suggested using simulation data where the direct N^+^(amine)...Asp1025-His1026-Cu3052(T1)-Cys1021-His1022-Cu3048(cluster) and conserved water (W1/ W2) mediated N^+^(amine)...Asp1025-His1026-Cu3052(T1)-Met1031-Glu1032...W1/W2...Cu3047(cluster) interaction is shown. Thus, an insight on the chemistry of neurotransmitter binding to hCP is reported.

## Figures and Tables

**Table 1:** Interaction of the acidic/polar residues of ceruloplasmin and water molecules from the potential sites of biogenic monoamine (Serotonin, Norepinephrine and Epinephrine)during MD-simulation of enzyme-substrate complexes

Neurotransmitter (Substrate)		Active site residues interacting with the substrate molecule (atoms). Distances are given in Å unit.						Residues/ Conserved
								Water center
								(Occupation
IUPAC (general)	Interacting atom	Time (ns)						frequency in %)
Name		5	10	15	20	25	30	
3-(2-Aminoethyl)-1	N1 (Amino nitrogen)	Glu272OE2	Glu272OE1	Glu272OE1	Glu935OE1/OE2	Glu935OE1/OE2	Glu935OE1	Glu272 (∼50)
H-indol-5-ol (Serotonin)		-2.68	-2.65	-2.52	(3.08/2.54)	3.10/2.86	2.71	Glu935 (∼50)
		Asp1025OD2	Asp1025OD2	Asp1025OD2	Asp1025OD2	Asp1025OD2	Asp1025OD2	Asp1025 (∼100)
		-2.74	-2.75	-2.75	-2.59	2.67	2.67	
		W2326	W2326	W2056	W2056	W2059	W2283	WS1 (∼100)
		-2.9	-2.7	-2.73	-3.03	2.67	2.89	
	N6 (Indole ring nitrogen)	-	W2309	W2309	W2295	W2295	W2295	WS2 (∼85)
			-2.79	-2.84	-2.62	2.66	2.75	
	O10 (5-hydroxyindole)	Asn271ND2	Asn271ND2	Asn271ND2	Asn271ND2	Asn271ND2	Asn271ND2	Asn271 (∼60)
		-3.1	-3.37	-3.41	-3.98	4.31	3.63	
		W2329(2.77)	W2329(2.73)	W2329 (2.69)	W2329(2.86)	W2329	W2329	WS3 (∼100)
					W2309(3.11)	-2.49	-2.67	
								
4-(2-amino-1-hydroxyethyl)	N1 (Amino nitrogen)	Asp206OD2	Glu272OE2	Glu272OE2	Glu272OE2	Glu272OE2	Glu272OE2	Asp206 (∼16)
benzene-1,2-diol (Norepinephrine)		-2.58	-2.72	-2.6	-2.58	-2.53	-2.51	Glu272 (∼84)
		Asp1025OD2	Asp1025OD1	Asp1025OD1	Asp1025OD1	Asp1025OD1	Asp1025OD1	Asp1025 (∼95)
		-3.62	-2.69	-2.6	-2.56	-2.49	-2.56	
		W2329	W2208	W2208	W2208	W2208	W2208	WN1 (∼100)
		-2.91	-2.74	-2.72	-3.81	-2.79	-2.65	
		-	-	-	W2211			
					-3.48			
	O3 (Benzylic hydroxyl)	D1025OD2	Glu272OE2	W2326	Glu272OE2	Glu935OE2	Glu935OE2	Asp1025 ∼16)
		-2.96	-2.5	-2.84	-2.58	-2.92	-2.62	Glu272 (∼35)
								Glu935 (∼30)
	O7 (para-hydroxyl of	Glu232OE2	Glu232OE1	Glu232OE2	Glu232OE2	Glu232OE2	Glu232OE2	Glu232 (∼100)
	benzene ring)	-2.71	-2.57	-2.67	-2.55	-2.51	-2.53	
	O8(meta-hydroxyl of	-	Glu232OE1	Glu232OE2	Glu232OE2	Glu232OE2	Glu232OE2	Glu232 (∼98)
	benzene ring)							
			-2.57	(2.71	-2.86	-2.82	-2.55	
4-(1-hydroxy-2-(methylamino)	N1(Amino nitrogen)	Asp1025OD1/OD2	Asp1025OD1/OD2	Asp1025OD2	Asp1025OD1/OD2	Asp1025OD1/OD2	Asp1025OD!/ODD2	Asp1025 (∼100)
ethyl)benzene-1,2-diol (Epinephrine)		(2.56/3.41)	(3.10/2.90)	-2.66	(3.03/2.65)	(3.28/2.90)	(3.20/2.56)	
		W2329	Glu935OE1	Glu935OE1/OE2	Glu935OE1/OE2	Glu935OE1/OE2	Glu935OE1	Glu935 (∼84)
		-2.83	-2.62	(3.09/2.68)	(3.22/2.60)	(3.32/2.61)	-2.57	
	O3(Benzylic hydroxyl)	Asp1025OD2	Glu935OE1	Glu935OE2	Glu935OE2	Glu935OE2	Glu935OE1	Asp1025 (∼16)
		-2.76	-2.7	-2.64	-2.75	-2.75	-2.76	Glu935 (∼84)
		W2051	-	-	-			
		-2.89						
	O7(para-hydroxyl of	Asp230OD2	Asp230OD1	Asp230OD1	Asp230OD1	Asp230OD1	Asp230OD1	Asp230 (∼100)
	benzene ring)	-2.65	-2.63	-2.61	-2.72	-2.76	-2.5	
		W2206	W2210	W2057	W2051	W2206	W2063	WE1 (∼100)
		-3.16	-2.95	-2.74	-3.09	-3.14	-2.91	
				W2052				
				-3.29				
	O8(meta-hydroxyl of	Asp230OD2	Asp230OD1	Asp230OD1	Asp230OD2	Asp230OD1	Asp230OD1	Asp230 (∼100)
	benzene ring)	-2.57	-2.71	-2.55	-2.55	-2.59	-2.7	
		-	-	Gln235NE2	W2063	Gln235NE2	Gln235NE2	Gln235 (∼60)
				-3.2	-3	-3.2	-3.45
